# Shellac Gum/Carrageenan Alginate-Based Core–Shell Systems Containing Peppermint Essential Oil Formulated by Mixture Design Approach

**DOI:** 10.3390/gels7040162

**Published:** 2021-10-03

**Authors:** Andrea Foglio Bonda, Alessandro Candiani, Martina Pertile, Lorella Giovannelli, Lorena Segale

**Affiliations:** Department of Pharmaceutical Sciences, Università del Piemonte Orientale, 28100 Novara, Italy; andrea.fogliobonda@uniupo.it (A.F.B.); alessandro.candiani@uniupo.it (A.C.); 20010990@studenti.uniupo.it (M.P.); lorella.giovannelli@uniupo.it (L.G.)

**Keywords:** alginate, shellac gum, carrageenan, encapsulation, core–shell system, peppermint essential oil, mixture design

## Abstract

Peppermint essential oil is encapsulated by inverse ionotropic gelation in core–shell systems, composed of alginate (ALG) alone or alginate with shellac gum (SHL) and/or carrageenan (CRG). A mixture design approach is used to evaluate the correlation between the formulation composition and some properties of the final products. Immediately after the preparation, capsules appear rounded with a smooth and homogeneous surface, having a similar particle size ranging from 3.8 mm to 4.5 mm. The drying process, carried out at 40 °C in an oven for 3 h, reduces capsules’ diameters by at least 50% and has a negative impact on the shape of the systems because they lose their regular shape and their external membrane partially collapses. The peppermint essential oil content of dried capsules is between 14.84% and 33.75%. The swelling behaviour of the systems is affected by the composition of their outer shell. When the external membrane is composed of alginate and shellac gum, the capsule ability to swell is lower than that of the systems containing alginate alone. The swelling ratio reaches 31% for alginate capsules but does not exceed 21% if shellac is present. Differently, when the second polymer of the shell is carrageenan, the swelling ability increases as a function of polymer concentration and the swelling ratio reaches 360%. In the case of systems whose outer membrane is a polymeric ternary mixture, the swelling capacity increases or decreases according to the concentrations of the individual polymers. The obtained results suggest that carrageenan could be a useful excipient to increase the swelling behaviour of the systems, while shellac gum makes the system shell more hydrophobic. The use of a mixture design (i.e., the use of ternary diagrams and related calculations), in which each single component is chosen to provide specific properties to the final mixture, could be the right approach to develop improved formulations with a tailored essential oil release profile.

## 1. Introduction

Core–shell systems are composed of an inner core (solid, liquid or gas) and of one or more outer layers. The shell is usually solid and composed of organic or inorganic materials, according to the final application of the resulting product and the selected production method. These systems can be obtained according to different technologies, for example, one-step straightforward electrospraying [1] and electrospinning [2], ionotropic gelation using a concentric nozzle [3] or inverse ionotropic gelation [4]. Core–shell systems find application in many fields such as the pharmaceutical, cosmetic and food industry, biomedical and material science and their characteristics are designed and defined according to the specific needs of the sector in which they are used [5]. These systems could be ideal to entrap (i.e., encapsulate) volatile substances or light or oxidation sensitive components, for example, essential oils, in order to protect them from the environmental impairment and/or to control their release, opening new possibilities to their practical uses. Moreover, the shell may include several different layers or a single layer composed of a mixture of different materials leading to obtain properties not achievable separately by a single component.

Several techniques can be adopted to encapsulate oil in general or essential oils, such as in situ polymerization, spray drying, solvent evaporation, self-assembly, ionotropic gelation, etc. [6,7,8]. In particular, inverse ionotropic gelation could represent a useful method to obtain mononuclear core–shell beads, in which the core is an oily liquid. To produce these beads, the core material is pre-mixed with divalent cations (usually Ca^2+^ ions) and the resulting solution/emulsion is dripped into an aqueous alginate solution [9,10,11]. Upon contact with the polymeric solution, divalent cations cross-link the guluronic group of the alginate chains to form a continuous shell around the liquid core. However, this technology requires the setting of several process and formulation parameters to guarantee the obtainment of a final product with appropriate characteristics. For example, as reported by Martins et al. [4], depending on the distance between the dropping nozzle and the gelling bath, the emulsion drops could assume different forms that influence the final capsule shapes. The ideal distance recommended to obtain regular in shape capsules is between 8 and 10 cm. Moreover, the viscosity of the core material plays an important role because it imparts resistance to the falling drop to win the forces during impact with the gelation bath and to reduce their deformation [6,12].

Lastly, the composition of the shell is also crucial in the definition of the final system properties; in many research papers, alginate is used in combination with other polymers in order to improve the characteristics of the beads [13] or to optimize the encapsulation efficiency, to change the release profile of encapsulated substances and the physicochemical properties of the capsules [14,15]. Shellac gum is the purified product of a natural lac; it is the resinous secretion of the parasite insect Kerria lacca on several species of Asian trees. It is recognized as safe in the FDA “Inactive Ingredients Guide” and is an approved food additive (E904) according to annex II/1333. This polymer finds application in oral drug delivery systems such as coating agent, in food products and cosmetic formulations [16,17,18,19]. Carrageenan is a family of high molecular weight sulphated polysaccharides obtained by the extraction of red seaweeds. It is composed of galactose and anhydrous galactose units linked by glycosidic unions [20]. In the food industry, it is used as a gelling, thickening, emulsifying and stabilizing agent. It is also a cosmetic ingredient selected for the formulation of toothpaste, air freshener gels, creams, shampoo, etc. Nowadays, carrageenan is also present in the pharmaceutical field; indeed, this polymer is employed in the formulation of oral extended-release tablets, as a carrier material in the formulation of pellets, microparticles and nanoparticles and as a viscosity enhancer [21,22].

Mixture design is a class of DoE (Design of Experiments) based on response surface methodology (RSM) that plots a response variable as a function of the different proportion of a mixture composed of different ingredients (generally, but not limited to, three components). This is an efficient strategy for the determination of the proportions of variables (ingredients) in a blend and their effect on determining the response variable [23]. For example, this approach was used in the pharmaceutical area by Foglio Bonda et al. [24] to understand the role of the formulation to obtain nanonized itraconazole powders, and it finds application also in the food field [25,26]. In the literature, there are examples proposing the use of statistical instruments for the optimization of process and/or formulation variables [27,28]. Nevertheless, there is a lack in the use of mixture design for the formulation design of beads or capsules produced by inverse ionotropic gelation.

The aim of this study is to develop oily core–shell systems by inverse ionotropic gelation using natural polymers as shell materials: alginate combined with shellac gum and/or carrageenan. Peppermint essential oil was selected as the oily phase in the core, while natural polymers were identified as useful materials for the shell composition because they result to be convenient and versatile for industrial applications. In the literature, there are studies in which shellac gum/alginate and carrageenan/alginate systems were proposed to produce composed carriers able to deliver and release drugs in a rate-controlled and targeted manner [11,29,30], but there are no references in which these polymers were used in a ternary combination. The mixture design approach is identified as a useful tool to study the influence of the composition of the formulation, in particular of the shell of the systems, on the properties of the final products.

## 2. Results and Discussion

### 2.1. Preliminary Studies

Core–shell systems with a peppermint essential oil core were produced by inverse ionotropic gelation. The preliminary studies supported the choice of locust bean gum (0.5% w/w) as a stabilizer of the emulsion dripped in the gelling baths. The other excipients tested as emulsion stabilizers were rejected for different reasons: arabic gum, gelatine and rice starch were not able to stabilize the emulsion as well as locust bean gum did when used at a low concentration; carrageenan made the emulsion too viscous and difficult to process, while the interaction of sodium caseinate with calcium ions was responsible for its precipitation. Locust bean gum at a 0.5% w/w concentration was enough to stabilize the emulsion avoiding a separation phase and leading to a more controlled release of bivalent ions responsible for the formation of the alginate shell and the production of capsules satisfying from a morphological point of view. Higher concentrations of this excipient in the emulsion caused the formation of capsules characterized by a non-homogeneous internal core (data not reported).

### 2.2. Mixture Design: Constraints and Feasible Region Definition

A mixture design was used to plan the proportions of the shell components (alginate, shellac gum and carrageenan) reported in Table 1.

The maximum limit for the ALG/SHL ratio useful to obtain capsules was found to be 0.25. In general, it is known that in the presence of calcium ions, shellac gum does not reticulate, but precipitates [11,31]. At a high amount of shellac gum in the mixture, this precipitate probably interferes with the alginate egg-box formation.

For carrageenan, the limit of ALG/CRG proportion was found to be 1.25. When carrageenan exceeded this limit, an increase in the viscosity of the gelation bath occurred; thus, compromising the core–shell system formation. In this work, iota-carrageenan was used; this is a highly sulphated natural polymer consisting of alternating residues of 3-linked β-d-galactopyranose and 4-linked 3,6-anhydro-α-pyranose. This polymer is characterized by well-known gelling properties when combined with divalent cations. Nevertheless, as reported by Gobet et al., carrageenan is also able to interact with monovalent cations resulting in an increase in viscosity of the polymer solution [32]. According to this assumption, a possible interaction between carrageenan and sodium ions present in the gelling bath and derived from the use of sodium alginate could be hypothesized.

### 2.3. Correlations

**Figure 1:**
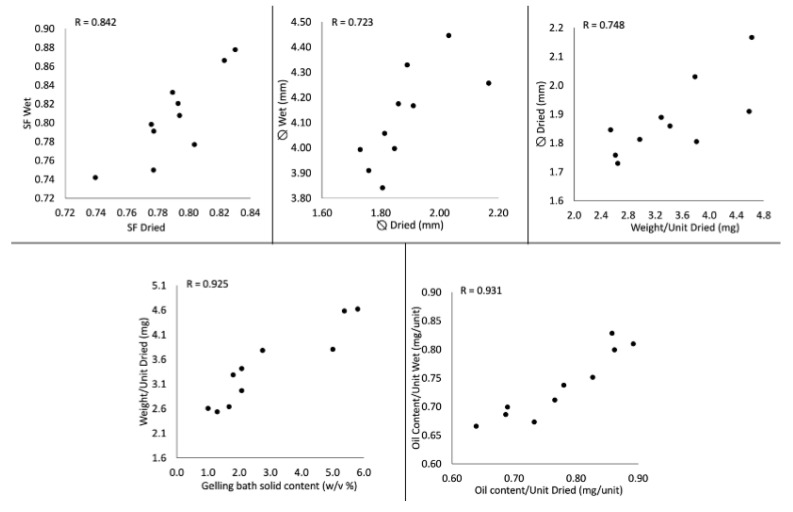
Correlation plots obtained by mixture design.

Five different correlations having R > 0.7 were found: this result can be considered an important indicator of the high correlation of these variables with each other. Figure 1 represents the plots of the aforementioned correlations. In three cases, a correlation was found inside the single response variable, referring to the production phases of the core–shell systems. In details, for the shape factor (R = 0.842), capsule diameter (R = 0.723) and capsule oil content (R = 0.931), a good correlation between the dried and the wet form of the systems was observed, suggesting that the drying phenomena had no impact on the aforementioned characteristics of the systems (i.e., capsules with the greatest diameter in the wet form were also the greatest in the dried form).

The other two correlations with R > 0.7 were the weight of the systems vs. the solid concentration of the gelation bath (R = 0.925) and the weight vs. the diameter of the systems in the dried state (R = 0.784). In the first case, the results agreed with those obtained in a previous work [11], where the heaviest beads were characterized by the greatest amount of excipients in the shell, while in the second case the correlation indicated that the density of the various polymeric shell mixtures after drying was quite similar.

### 2.4. Morphology and Dimensions

**Figure 2:**
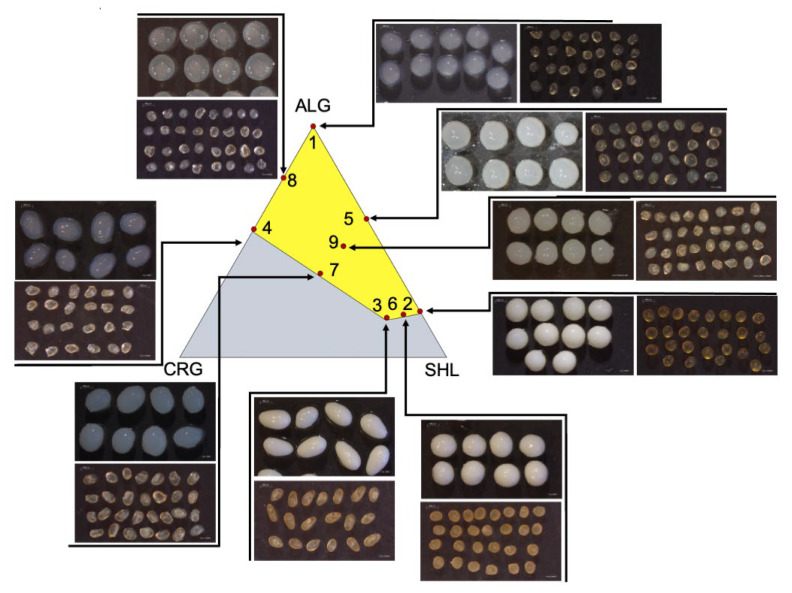
Experimental points and corresponding wet and dried core–shell systems.

Immediately after the preparation, capsules were rounded even if not perfectly spherical (shape factors, SF, were far from 1), with the exception of P3 and P7 batches which were slightly elongated (Table 2). All systems were characterized by a smooth and homogeneous surface, even if they appeared different in colour according to the various shell polymeric composition (Figure 2, Figures S1,2). In P1, P4 and P8 capsules, it was possible to clearly distinguish the presence of a core and of an external membrane surrounding it; otherwise, in the case of P2, P3, P5, P6, P7 and P9 systems, the opacity of the capsules made this distinction impossible to the naked eye. The colour was more intense and tending to be yellow for P2, P3 and P6 formulations, that is, those with the highest percentage of shellac gum. This was presumably attributable to the precipitation of the polymer induced by the interaction with the calcium ions present in the internal phase of the emulsion, as suggested by Messaoud et al. [31]. The capsule surface became shinier and the colour faded when the percentage of shellac gum decreased, as evidenced by the images of P7, P9 and P5 systems (Figure 2). After drying, core–shell systems became uniform in colour and decreased in dimensions (Figure 2). However, the drying process had a negative impact on the shape of the systems: they lost their regular shape and a partial collapse of their external membrane was evident.

Core–shell systems in the wetted state had similar particle sizes: their average diameters ranged from 3.8 mm to 4.5 mm (Table 2). The composition of the outer shell, its structural complexity and the interactions between the different polymer chains (alginate, shellac gum and carrageenan) defined the achievable dimensional limit. After drying, an evident contraction of volume and size of the capsules was observed, associated with a decrease in their diameters of at least 50% and attributable to the loss of water and the consequent packing of the polymeric chains constituting the structure of the systems (Table 2).

The variation of some morphological parameters (shape factor of the wet and dried capsules, the diameter of the dried capsules and their weight) as a function of mixture components resulted to be described by mathematical models reported in Table 3, Tables S1 and S2.

The shape factor (SF both for capsules in a dried and wet state) was described by a linear model, the diameter of the dried beads by a special cubic model, while a quadratic model was computed for the weight of the capsules in the dried form. In these cases, the adjusted R^2^ was comprised between 0.78 and 0.94 meaning that the models could describe the response variable variations with a good agreement; in contrast, the prediction capability (Predicted R²) needs to be further improved, especially for variables such as the diameter of capsules in the dried form; however, data prediction was not the main focus of this paper.

**Figure 3:**
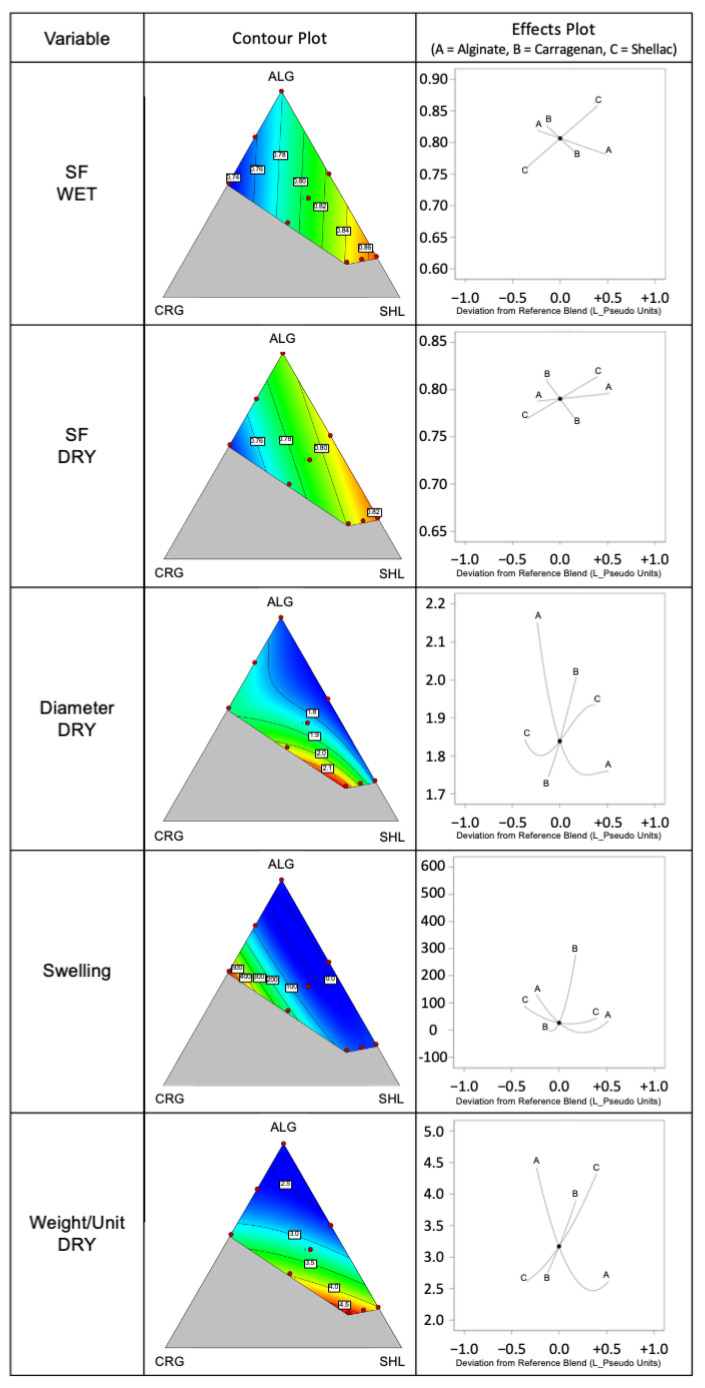
Contour plots and effect plots of significative models.

In Figure 3, for the significative models, the contour plot and the relative effect plot are shown. It was possible to observe how the shape factor (both for capsules in the wet and dried form) linearly increased when high amounts of shellac gum were included in the mixture. On the contrary, carrageenan negatively affected this response variable in a linear way as showed in the effect plot. Carrageenan, by its nature, is an excipient that increases the viscosity of water-based formulations [32], so it also played this role when included in the composition of the gelling bath. As a result, an increase in the gelling bath viscosity led to the production of capsules with large deformations and characterized by low shape factor values [12].

Considering that the composition and the weight of the dripping phase were the same in all experiments and that a drop weighed about 10 mg (10.9 ± 1.2 mg, average of 10 determinations), the weight of the capsules could change only as a function of the composition of the gelation bath, in particular as a function of its solid concentration. From the effect plot, it was challenging to select the components that had the main impact on the weight of the capsules. This variable increased moving from the bath composed of a single polymer (alginate, 1% solid concentration) to those composed of the polymeric mixtures with the highest amounts of both shellac gum and carrageenan (P3, 5.80% and P6, 5.37% solid concentration). It was clear that when all the systems had the same volume in the wet state, their weight became different after water evaporation if the initial solid concentration was different.

The drying process was responsible for capsule size reduction and the formulations characterized by the lowest diameter decrease were P2 (52.86%), P3 (49.00%) and P6 (54.19%), those with an important concentration of solid in the gelation bath. In this case, the high solid quantity probably sterically impeded the contraction of the polymeric structure during drying, the tight packing of the polymer chains and an important reduction in the capsule diameter. This trend was evidenced in the relative contour plot (Figure 3), where the behaviour of this variable was similar to that of the variable weight of the capsules.

### 2.5. Essential Oil Content

The peppermint essential oil content in freshly prepared core–shell systems (wet capsules) was between 1.66% and 2.25% and, after drying, because of water evaporation, this value increased to 14.84% and 33.75%. The essential oil content of the dried P2, P3 and P6 formulations was lower than that of all the other systems despite the fact that the essential oil content per unit, expressed in mg, was comparable (Table 2). This was justified by the fact that, as it was evident from the data reported in Table 2, the weight of P3 and P6 dried capsules (between 4.5 mg and 4.6 mg) was higher compared to that of the other formulations (between 2.5 mg and 3.8 mg). The explanation of the highest weight of P2, P3 and P6 units was related to the solid content in the gelling bath during the preparation step which exceeded 5% (Table 1).

In these cases, higher amounts of polymers took part in the external shell formation causing an increase in the weight of the resulting capsules. For this reason, in Table 2, the essential oil content per unit was reported instead of the oil loading percentage, that was strongly affected by the capsules weight. No significant reduction in the essential oil content per unit was detected after drying, indicating the ability of the polymeric structure of the capsules to avoid the evaporation of essential oil. Moreover, comparing the essential oil content per drop of the dripping emulsion (0.72 mg ± 0.09 mg) with the essential oil mg per unit reported in Table 1 and relative to wet and dried capsules, it was possible to observe that the composition of the shell did not affect the encapsulation efficiency both during the gelation and drying phases, because a complete oil encapsulation was reached in all the experiments.

### 2.6. Swelling

When the dried alginate capsules were put in contact with an aqueous fluid, they were able to rehydrate and swell, as water molecules penetrated into the system, causing a spacing of polymer chains and leading to an increase in the volume. The swelling extent depends on many factors such as the characteristics of the sample and the nature of the fluid with which these systems come into contact with [33].

**Figure 4:**
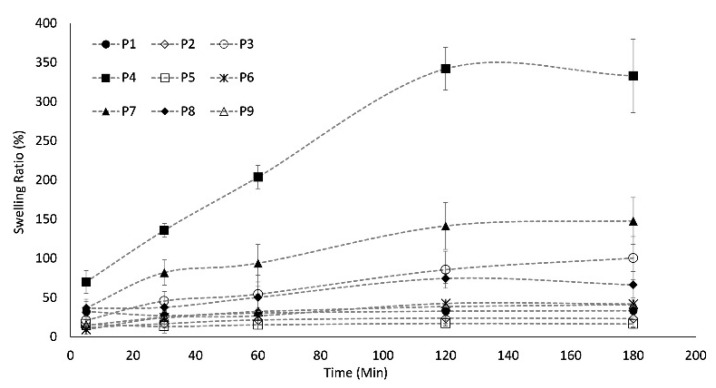
Swelling profiles of the systems.

Comparing the swelling ability of all the formulations, a first analysis of the results evidenced that the composition of the outer shell of the systems had an important impact on this behaviour. In particular, when the external membrane was composed of a combination of alginate and shellac gum, the capsule attitude to swelling was lower than that of the systems containing alginate alone (P1). In detail, after only a few minutes in the fluid, P1 samples showed a more marked swelling (the swelling ratio reached 31%) compared to the P5 and P2 systems (21% and 13% of swelling ratio, respectively) (Figure 4).

Even in the successive steps of the test, the tendency of shellac gum systems to swell was more contained than that of the reference (P1), to indicate the predisposition of shellac gum to prevent the penetration of fluids into the system. The presence of this polymer probably created a rather tight shell structure that opposed water uptake, limiting the swelling capacity of the systems. The literature supports this theory: shellac gum is known for its ability to modify the drug release rate and it was successfully used in oral controlled release dosage forms such as pellets produced by a fluid bed coating process [17].

If the polymeric binary mixture included in the shell was formed by carrageenan and alginate, the swelling properties of the resulting systems were certainly more remarkable than those of the reference capsules (P1). Indeed, when the second polymer of the shell was carrageenan, for example in the P4 and P8 systems, the swelling ability increased as a function of carrageenan concentration (Figure 4): the capsules in which this polymer was present in a low percentage (P8) reached a 79% swelling ratio after 120 min of contact with the fluid. This value was also gained by the P4 formulation (high carrageenan percentage content) but, if in the case of P8 it represented the maximum achievable swelling ratio, then in the case of the P4 system it indicated only the swelling percentage after a few minutes of exposure to the fluid. In fact, after only 5 min of contact with water, the P4 system reached about 80% of swelling and after 120 min overcame 360%.

Finally, in the case of systems whose outer membrane was a ternary mixture of alginate, carrageenan and shellac gum, the positive or negative effect in terms of increasing or decreasing the swelling capacity was closely related to the concentrations of the individual polymers.

A quadratic model was computed for the description of the swelling behaviour of the capsules in the dried form (Table 3). The swelling values at 180 min were computed as a response variable in the mixture design, obtaining the relative plots (Figure 3). The effect plot shows an exponential increase in the swelling ratio towards the highest amount of carrageenan in the formulation, while the other secondary polymer of the shell (shellac gum) did not contribute to increase the swelling behaviour of the systems. The carrageenan effect was evident both in the P4 (shell composed of alginate and carrageenan) and in P7 systems, where alginate, carrageenan and shellac gum were present: carrageenan played the main role in modifying the swelling ability of the capsules and its effect was directly related to the amount of this component in the formulation. In details, when the carrageenan percentage overcame a critical limit (about 25% w/w), its effect became prevalent, causing a dramatic increase in the swelling ratio. This swelling ability of kappa-carrageenan was previously reported by Mohamadnia et al. [34] and Wang et al. [35], suggesting that the presence of sulphate groups promotes an important swelling ability in hydrogel networks. These results confirmed that both kappa- and iota-carrageenan could be used as drug release modulators in the formulation of solid dosage forms according to their swellable properties.

### 2.7. In Vitro Essential Oil Release Profile

This test was conducted on dried P1, P2 and P4 capsules in a phosphate buffer at pH 6.8 with the addition of 96% ethanol in order to evaluate the ability of different core–shell systems to release the essential oil. These formulations were selected after the mixture design analysis, which indicated them as the most representative formulations of different behaviours in the design space (e.g., P4 characterized by the greatest swelling ratio, P2 as the formulation with the highest shape factor and P1 as reference). In this way, it was possible to evaluate how the different composition of the external shell, consisting of alginate alone (P1) or of a binary mixture of alginate and carrageenan (P4) or shellac gum (P2), could influence the essential oil release.

**Figure 5:**
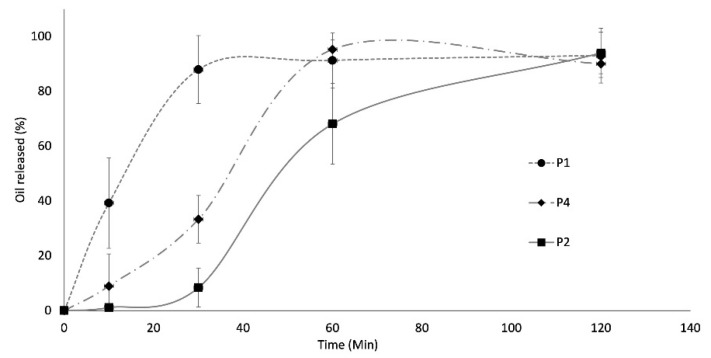
In vitro essential oil release profile of the systems.

As expected, the trend of the release profiles varied according to the external shell composition, but all the formulations did not show any initial burst-effect, meaning that the essential oil was not on the surface of the capsules. When alginate was the only component of the shell, the release process started rapidly and after only 30 min from the beginning of the test, 90% of the loaded essential oil was released (Figure 5). On the other hand, the combination of alginate and carrageenan (P4) gave rise to a system which required more time to conclude the oil release process. The important swelling predisposition of carrageenan probably caused the rapid formation of a gel layer on the capsule’s surface, which slowed down the release of the essential oil. In this case, after 30 min from the start of the test, the percentage of oil in the ethanolic solution did not overcome 40%, and the process ended in about two hours (Figure 5). In the case of P2 formulation, the role of shellac gum was evident: the presence of this polymer reduced the affinity of the system for the aqueous fluid, limited its swelling ability and opposed the release of the essential oil. In this case, the release profile was typical of a biphasic process: it was characterized by an initial lag-time, during which the oil release was rather slow (after 30 min, no more than 12% of the loaded oil was released from the system), followed by a phase during which the oil was released more quickly (Figure 5). During the first phase of the release process, the containment effect of the shellac gum was probably dominant and the essential oil was unable to diffuse through the polymeric structure of the shell, while this effect disappeared as the process went on.

## 3. Conclusions

The production of core–shell systems by inverse ionic gelation having different shell compositions (up to three components) was possible. The technique was effective to encapsulate liquid substances with high recovery regardless of the composition of the shell. As expected, shell composition had a great impact on some technological characteristics of the final products and the mixture design approach represented a useful tool to identify the optimal composition of the formulation, in order to obtain a final product with the required characteristics. In this study, the effects of two natural polymers (shellac gum and carrageenan) in addition to alginate in the development of bio-based solid core–shell systems loaded with essential oil were investigated. The obtained results suggest that carrageenan could be a useful excipient to increase the swelling of the systems, while shellac gum made their shell more hydrophobic. The use of the mixture design approach, to guarantee specific properties of the final mixture associated with each single component’s contribution, could be the right approach for the development of ameliorated formulations with tailored essential oil release profiles.

## 4. Materials and Methods

### 4.1. Materials

Peppermint essential oil (containing L-Menthol and trans-p-Menthan-3-one as main components) was kindly donated by FREY&LAU GmbH (Henstedt-Ulzburg, Germany); sodium alginate (ALG) (molecular weight 120,000–190,000 g/mol; ratio of mannuronic-guluronic 1.56), calcium chloride was purchased from Sigma-Aldrich (Milan, Italy). Locust bean gum (Ceratonia Siliqua Flour Seeds) was purchased from Farmalabor (Milan, Italy). Shellac gum (SHL, SSB 55 Pharma FL) was kindly donated by Stroever Schellack Bremen (Bremen. D) and iota-carrageenan (CRG, GENUVISCO CG-131) by Giusto Faravelli S.p.A (Milan, Italy). All other reagents were of analytical grade and used as received.

### 4.2. Experimental Methods

#### 4.2.1. Preliminary Studies

Preliminary studies were performed in order to select the best core capsule composition: different formulations of dripped emulsion were evaluated in terms of stability and capsule morphology with the aim to select the best emulsion stabilizer (type and concentration). Taking that into consideration, in a previous work, hydroxyethylcellulose (HEC) was used as emulsion stabilizer of the dripped emulsion with satisfying results [11]; the goal of these preliminary studies was to substitute HEC with a natural compound able to guarantee the same stabilizing efficacy, avoiding the use of synthetically derived substances. Locust bean gum, arabic gum, gelatine, carrageenan, rice starch and sodium caseinate were identified as possible candidates and used in a range of concentrations between 0.5 and 15.0% w/w according to the specific stabilizing properties of each substance.

In a CaCl_2_ aqueous solution (1.35 M), the predefined percentage of different stabilizers was added under magnetic stirring. After complete solubilization of the stabilizer, peppermint essential oil was added (7.5% w/w), and the resulting emulsion was maintained under magnetic stirring (1500 rpm) for 30 min. The different emulsions were transferred into a glass tube, maintained at room temperature for 16 h, visually inspected at predefined time intervals, and compared to emulsion with HEC used as reference (data not reported). Locust bean gum (0.5% w/w) was able to stabilize the emulsion and to replicate HEC behaviour, and for this reason it was selected as stabilizer for the next experiments.

#### 4.2.2. Capsules Preparation

The aforementioned core formulation (CaCl_2_ solution 1.35 M, peppermint essential oil 7.5% w/w and locust bean gum 0.5% w/w) was used to prepare core–shell capsules by inverse ionotropic gelation. The emulsion was dripped into the gelling bath through an 800 µm in diameter needle, maintained at 10 cm above the surface of the bath. The emulsion droplets fell into a polymeric aqueous solution containing alginate or alginate in combination with shellac gum and/or carrageenan.

After 2 min of curing, wet capsules were filtered, washed with deionized water and transferred in CaCl_2_ solution (100 mM) for 10 min. Successively, the capsules were recovered from CaCl_2_ solution, rinsed with deionized water and dried in an oven at 40 °C for 3 h. The gelation time was fixed to 2 min because after this period the forming capsules began to coagulate with each other.

All the capsule formulations were prepared with similar procedure but varying the gelling bath composition. In details, the gelling bath was a 1% w/w alginate aqueous solution enriched by a secondary excipient (shellac gum or carrageenan) or a mixture thereof in a defined proportion according to the mixture design approach (Table 1). The gelling bath was prepared as follows: sodium alginate powder was dissolved in deionized water under stirring to obtain the required concentration; carrageenan was then added to the polymeric solution. For shellac gum containing formulations, the gelling bath was prepared according to the method reported by Messaoud et al. [31]: shellac gum (5% w/w) was solubilized in 0.5% w/w ammonium carbonate solution at 50 °C; the obtained solution was heated to 60 °C until a constant pH, indicative of the complete elimination of the ammonium salt excess, was reached. After replacing the water loss, shellac gum solution was diluted with alginate or alginate/carrageenan solution until the required concentration was obtained.

#### 4.2.3. Mixture Design

A mixture design was used to plan the proportions of the shell components (alginate, shellac gum and carrageenan) and the data collected from the characterization of core–shell systems were used as response variables.

##### Constraints and Feasible Region Definition

**Figure 6:**
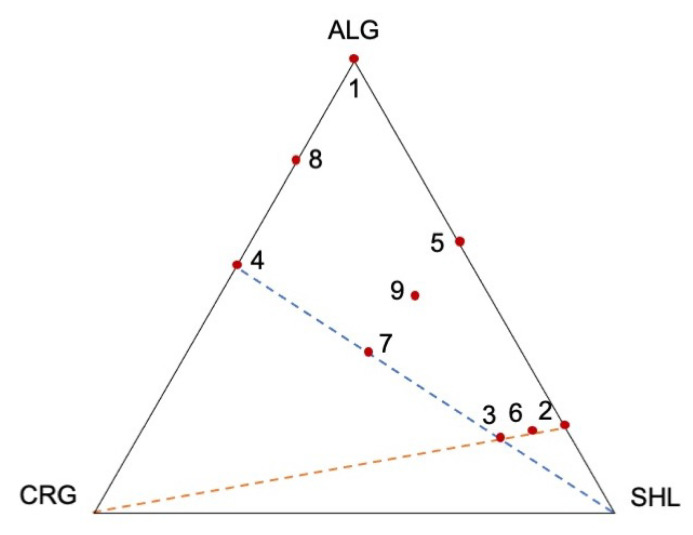
Constraints and experimental points definition.

In order to define the experimental points, some screening experiments were performed to understand the mixture area in which it was possible to produce regular capsules by inverse ionotropic gelation. Taking into consideration that, when alginate was the only component of the shell, it was well known that it was possible to obtain satisfying final products [9], alginate amount in the gelation bath was set at 1% w/v and the limits of the other two polymers were investigated. Preliminary tests were performed and, here, were briefly summarized: placebo systems with the shell constituted by a binary mixture of alginate and shellac gum or alginate and carrageenan were produced, increasing, at each experiment, the amount of the secondary polymer until it was possible to obtain well separated and well-formed capsules. When shellac gum was greater than 80% w/w (alginate/shellac gum ratio ≤ 0.25), the resulting systems were too weak and disintegrated immediately under a magnetic stirring force. For this reason, the proportion limit between alginate and shellac gum (i.e., alginate/shellac gum ratio) was set at 0.25 and a line between the point ALG:SHL 20:80 and the opposite vertex of the domain (ALG:SHL:CRG = 0:0:100) was drawn, as reported in Figure 6.

The carrageenan limit selection gave ALG/CRG a proportion of 1.25, that is, alginate 55.5% w/w and carrageenan 45.5% w/w, and, in this case, the limit was dictated by the gelation bath viscosity because, when the carrageenan concentration was higher than 45.5% w/w, it was too high and responsible for elongated and “worm like” capsules. A line between the point ALG:CRG 55.5:44.5 and the opposite vertex of the domain (ALG:SHL:CRG = 0:100:0) was drawn (Figure 6). Based on the aforementioned constraints, a polygonal sub-region was identified, and 9 experimental points were selected by the most informative approach used by Foglio Bonda et al. [24]: 4 experimental points at the vertexes of the polygon, 4 at the middle points of its edges and 1 as the centroid. The proportions of the mixtures are reported in Table 2. The centroid experiment (P9) was replicated in order to understand the system variability, so a total of 10 experiments were carried out.

##### Statistical Analysis

A correlation matrix was created between all the response variables in order to evaluate any relationship, whether causal or not, among them. In addition to the correlation matrix, an additional parameter was added, that is, the theoretical solute concentration of the gelation bath.

For each response variable, a multivariate regression analysis was performed in order to select the best model that fit with the mixture variation using a forward selection procedure based on the Akaike information criterion corrected for small sample sizes (AICc). For the significative model, a contour plot (response surface) and an effect plot were generated. Design Expert^®^ software (version 12) was used for all statistical analyses, no experimental data transformation (e.g., Log. or Exp.) was necessary because the software worked in “pseudo” coordinates by itself [24].

#### 4.2.4. Characterization of Core–Shell Systems

##### Morphology and Dimensions

Size and shape of the capsules were investigated using optical microscopy (Stereomicroscope Leica S9i) immediately after the preparation (in the swollen state, wet capsules) and after the drying process in an oven at 40 °C for three hours (in the dried state, dried capsules).

Mean diameter was determined by image analysis (ImageJ software, National Institute of Health, Bethesda, MD, USA) [36] and calculated as the average between maximum and minimum diameter of each particle considered, and the shape factor (SF) was defined according to the equation reported below:

SF = 4πA/P^2^ (1)

where A is the area and P is the perimeter of the particles. This parameter could vary from 0 to 1 and 1 was the result obtained in the case of a very regular in shape capsule, namely, for a capsule with a circular projection.

Dimensions and shape factors were determined on at least 50 samples for each batch.

##### Essential Oil Content

Essential oil content was determined on the core–shell systems immediately after the preparation and after the drying process using an UV spectrophotometric method. Three wet or dried capsules were exactly weighed and dispersed in 1 mL phosphate-buffered solution at pH 6.8, and maintained under vigorous stirring. After 1 or 2 h, depending on the formulation composition, the complete disaggregation of the core–shell systems was reached and 9 mL ethanol was added in order to promote alginate precipitation and the solubilization of essential oil in the alcoholic phase. Then, the dispersion was filtered through 0.45 µm filters and the obtained solution analysed at 260 nm wavelength. Peppermint essential oil content was calculated through the calibration curve previously constructed using essential oil at different known concentrations (between 0.09 and 1.56 mg/mL; R^2^ = 0.998) in phosphate buffer–ethanol solution (1:9 ratio).

The essential oil content was expressed as milligrams of oil for single unit and the results were the average of three determinations. The essential oil content of the dripping emulsion was determined and used as control.

##### Swelling Studies

Swelling studies in deionized water were performed on dried capsules. The ability of the core–shell systems to absorb the fluid was determined by weight: for each formulation, 5 dried particles (corresponding to about 0.2 g) were weighed and introduced into a vial in which 5 mL of fluid maintained at 37 °C was added. The vial was placed in a thermostatic bath at 37 °C and, at predefined time intervals (5, 30, 60, 120 and 180 min), samples were recovered and weighed again. The swelling percentage was calculated according to the equation reported below:

Sw % = 100 *x* (W_t_ − W_0_)/W_0_ (2)

where W_t_ is the weight of the core–shell systems in the swollen state at time t and W_0_ is the initial weight of the dried systems [37]. The results were the average of three determinations.

##### In Vitro Essential Oil Release Test

The in vitro essential oil release tests were carried out on three formulations (P1, P2 and P4 of mixture design plan). For each batch, two exactly weighed capsules were put in 0.5 mL of phosphate buffer (pH 6.8) under stirring, and after predefined time intervals (10, 30, 60 and 120 min) 4.5 mL of ethanol was added. The dispersion was maintained under stirring for 40 min to favour the solubilization of the oil released by the capsules in the alcoholic phase. Successively, it was filtered (0.45 µm filters) to eliminate the capsules not yet completely disaggregated or the portion of alginate already solubilized in the buffer and precipitated after contact with ethanol. The alcoholic solution was analysed by UV (at 260 nm) to determine its oil content. The percentage of essential oil released after each time was calculated considering the initial capsule oil content. The results were the average of three determinations.

## Figures and Tables

**Table 1:** Composition of mixture design experimental points and solid content of each gelling bath.

Experimental Point	Mixture Composition (w/w%)			Gelling Bath Solid Content (w/v%)
	Alginate	Carrageenan	Shellac Gum	
P1	100.00	0.00	0.00	1.00
P2	20.00	0.00	80.00	5.00
P3	17.24	13.79	68.97	5.80
P4	55.56	44.44	0.00	1.80
P5	60.00	0.00	40.00	1.67
P6	18.62	6.90	74.48	5.37
P7	36.40	29.12	34.48	2.75
P8	77.78	22.22	0.00	1.29
P9	48.20	14.56	37.24	2.07

**Table 2:** Summary of the characterizations evaluated in the mixture design.

Exp. Point	Wet Capsules				Dried Capsules			
	SF ± SD	Diameter (mm ± SD)	Oil Content/Unit (mg ± SD)	Weight/Unit (mg ± SD)	SF ± SD	Diameter (mm ± SD)	Oil Content/Unit (mg ± SD)	Weight/Unit (mg ± SD)
P1	0.777 ± 0.066	3.91 ± 0.30	0.712 ± 0.04	35.78 ± 3.21	0.803 ± 0.065	1.76 ± 0.23	0.765 ± 0.01	2.61 ± 0.21
P2	0.866 ± 0.025	3.84 ± 0.21	0.700 ± 0.04	31.92 ± 2.39	0.822 ± 0.019	1.81 ± 0.18	0.689 ± 0.04	3.81 ± 0.08
P3	0.808 ± 0.052	4.26 ± 0.68	0.686 ± 0.14	41.31 ± 2.82	0.794 ± 0.033	2.17 ± 0.44	0.686 ± 0.09	4.62 ± 0.32
P4	0.742 ± 0.054	4.33 ± 0.24	0.800 ± 0.02	47.56 ± 3.86	0.739 ± 0.095	1.89 ± 0.45	0.861 ± 0.08	3.29 ± 0.25
P5	0.820 ± 0.020	3.99 ± 0.20	0.810 ± 0.03	39.76 ± 6.52	0.793 ± 0.043	1.73 ± 0.17	0.892 ± 0.08	2.64 ± 0.74
P6	0.878 ± 0.006	4.17 ± 0.24	0.738 ± 0.05	38.58 ± 1.48	0.830 ± 0.025	1.91 ± 0.16	0.780 ± 0.20	4.58 ± 0.15
P7	0.791 ± 0.040	4.45 ± 0.61	0.829 ± 0.04	39.01 ± 2.25	0.777 ± 0.061	2.03 ± 0.35	0.858 ± 0.03	3.78 ± 0.44
P8	0.750 ± 0.050	4.00 ± 0.35	0.673 ± 0.02	38.17 ± 1.42	0.777 ± 0.063	1.85 ± 0.24	0.732 ± 0.05	2.54 ± 0.09
P9	0.832 ± 0.045	4.18 ± 0.29	0.751 ± 0.03	33.32 ± 0.31	0.789 ± 0.062	1.86 ± 0.23	0.826 ± 0.04	3.41 ± 0.07

**Table 3:** Summary of significative mathematical models.

Variable	Model	*p*-Value	Adjusted R²	Predicted R²
SF WET	Linear	0.001	0.82	0.76
SF DRY	Linear	0.002	0.78	0.65
Diameter DRY	Special Cubic	0.031	0.88	0.42
Weight unit DRY	Quadratic	0.008	0.90	0.68
Swelling	Quadratic	0.002	0.94	0.55

## Supplementary Materials

The following are available online at https://www.mdpi.com/article/10.3390/gels7040162/s1, Figure S1: Detailed images of the wet core-shell systems (numbers correspond to the Experimental Points), Figure S2: Detailed images of the dried core-shell systems (numbers correspond to the Experimental Points). , Table S1: TModels and corresponding equation used in the statistical analysis, Table S2: Coefficients calculated for each significative model.

## References

[1] Loscertales G., Barrero A., Guerrero I., Cortijo R., Marquez M., Gañán-Calvo A.M. (2002). Micro/nano encapsulation via electrified coaxial liquid jets. Science.

[2] Ding Y., Dou C., Chang S., Xie Z., Yu D.G., Liu Y., Shao J. (2020). Core–shell Eudragit S100 nanofibers prepared via triaxial electrospinning to provide a colon-targeted extended drug release. Polymers.

[3] Choi D.H., Park C.H., Kim I.H., Chun H.J., Park K., Han D.K. (2010). Fabrication of core-shell microcapsules using PLGA and alginate for dual growth factor delivery system. J. Control. Release.

[4] Martins E., Renard D., Adiwijaya Z., Karaoglan E., Poncelet D. (2017). Oil encapsulation in core–shell alginate capsules by inverse gelation. I: Dripping methodology. J. Microencapsul..

[5] Ramli R.A., Laftah W.A., Hashim S. (2013). Core–shell polymers: A review. RSC Adv..

[6] Abang S., Chan E.S., Poncelet D. (2012). Effects of process variables on the encapsulation of oil in Ca-alginate capsules using an inverse gelation technique. J. Microencapsul..

[7] Mishra M. (2016). Handbook of Encapsulation and Controlled Release.

[8] Lopedota A.A., Arduino I., Lopalco A., Iacobazzi R.M., Cutrignelli A., Laquintana V., Racaniello G.F., Franco M., La Forgia F., Fontana S. (2021). From oil to microparticulate by prilling technique: Production of polynucleate alginate beads loading Serenoa Repens oil as intestinal delivery systems. Int. J. Pharm..

[9] Martins E., Poncelet D., Rodrigues R.C., Renard D. (2017). Oil encapsulation in core–shell alginate capsules by inverse gelation II: Comparison between dripping techniques using W/O or O/W emulsions. J. Microencapsul..

[10] Tsai F.H., Chiang P.Y., Kitamura Y., Kokawa M., Islam M.Z. (2017). Producing liquid-core hydrogel beads by reverse spherification: Effect of secondary gelation on physical properties and release characteristics. Food Hydrocoll..

[11] Foglio Bonda A., Regis L., Giovannelli L., Segale L. (2020). Alginate/maltodextrin and alginate/shellac gum core-shell capsules for the encapsulation of peppermint essential oil. Int. J. Biol. Macromol..

[12] Morales A.H., Spuches F.C., Hero J.S., Alanis A.F., Martinez M.A., Romero C.M. (2021). Impact of prosopis nigra gum exudate in alginate core-shell synthesis by inverse gelation technique. Food Hydrocoll..

[13] Leong J.Y., Lam W.H., Ho K.W., Voo W.P., Lee M.F.X., Lim H.P., Lim S.L., Tey B.T., Poncelet D., Chan E.S. (2016). Advances in fabricating spherical alginate hydrogels with controlled particle designs by ionotropic gelation as encapsulation systems. Particuology.

[14] Puttipipatkhachorn S., Pongjanyakul T., Priprem A. (2005). Molecular interaction in alginate beads reinforced with sodium starch glycolate or magnesium aluminum silicate, and their physical characteristics. Int. J. Pharm..

[15] Banerjee S., Singh S., Sankar S. (2013). Trivalent ion cross-linked pH sensitive alginate-methyl cellulose blend hydrogel beads from aqueous template. Int. J. Biol. Macromol..

[16] Buch K., Penning M., Wächterbach E., Maskos M., Langguth P. (2009). Investigation of various shellac grades: Additional analysis for identity. Drug Dev. Ind. Pharm..

[17] Farag Y., Leopold C.S. (2011). Development of shellac-coated sustained release pellet formulations. Eur. J. Pharm. Sci..

[18] Khorram F., Ramezanian A., Hosseini S.M.H. (2017). Shellac, gelatin and Persian gum as alternative coating for orange fruit. Sci. Hortic..

[19] Wang K., Wen H.F., Yu D.G., Yang Y., Zhang D.F. (2018). Electrosprayed hydrophilic nanocomposites coated with shellac for colon-specific delayed drug delivery. Mater. Des..

[20] Coviello T., Matricardi P., Marianecci C., Alhaique F. (2007). Polysaccharide hydrogels for modified release formulations. J. Control. Release.

[21] Campo V.L., Kawano D.F., Silva D.B., Carvalho I. (2009). Carrageenans: Biological properties, chemical modifications and structural analysis—A review. Carbohydr. Polym..

[22] Li L., Ni R., Shao Y., Mao S. (2014). Carrageenan and its applications in drug delivery. Carbohydr. Polym..

[23] Carmo A.C.M., Cunha-Filho M.S.S., Gelfuso G.M., Gratieri T. (2017). Evolution of quality on pharmaceutical design: Regulatory requirement?. Accredit. Qual. Assur..

[24] Foglio Bonda A., Rinaldi M., Segale L., Palugan L., Cerea M., Vecchio C., Pattarino F. (2016). Nanonized itraconazole powders for extemporary oral suspensions: Role of formulation components studied by a mixture design. Eur. J. Pharm. Sci..

[25] Kpodo F.M., Afoakwa E.O., Amoa B.B., Saalia F.K.S., Budu A.S. (2013). Application of multiple component constraint mixture design for studying the effect of ingredient variations on the chemical composition and physico-chemical properties of soy-peanut-cow milk. Int. Food Res. J..

[26] Buruk Sahin Y., Aktar Demirtaş E., Burnak N. (2016). Mixture design: A review of recent applications in the food industry. J. Eng. Sci..

[27] Politis S.N., Colombo P., Colombo G., Rekkas D.M. (2017). Design of experiments (DoE) in pharmaceutical development. Drug Dev. Ind. Pharm..

[28] Russo P., Zacco R., Rekkas D.M., Politis S., Garofalo E., Del Gaudio P., Aquino R. (2018). Application of experimental design for the development of soft-capsules through a prilling, inverse gelation process. J. Drug Del. Sci. Technol..

[29] Li L., Zhao J., Sun Y., Yu F., Ma J. (2019). Ionically cross-linked sodium alginate/ĸ-carrageenan double-network gel beads with low-swelling, enhanced mechanical properties, and excellent adsorption performance. Chem. Eng. J..

[30] Sayeesh P.M., Reshma J., Nibisha P., Franklin J., Jinu G. (2019). Alginate/k-carrageenan and alginate/gelatin composite hydrogel beads for controlled drug release of curcumin. Adv. Mater. Lett..

[31] Messaoud G.B., Sánchez-González L., Probst L., Jeandel C., Arab-Tehrany E., Desobry S. (2016). Physico-chemical properties of alginate/shellac aqueous-core capsules: Influence of membrane architecture on riboflavin release. Carbohydr. Polym..

[32] Gobet M., Mouaddab M., Cayot N., Bonny J.M., Guichard E., Le Quéré J.L., Moreau C., Foucat L. (2009). The effect of salt content on the structure of iota-carrageenan systems: 23Na DQF NMR and rheological studies. Magn. Reson. Chem..

[33] Mannina P., Segale L., Giovannelli L., Foglio Bonda A., Pattarino F. (2016). Self-emulsifying excipient platform for improving technological properties of alginate-hydroxypropylcellulose pellets. Int. J. Pharm..

[34] Mohamadnia Z., Zohuriaan-Mehr M.J., Kabiri K., Jamshidi A., Mobedi H. (2008). Ionically cross-linked carrageenan-alginate hydrogel beads. J. Biomater. Sci. Polym. Ed..

[35] Wang Y., Liu M., Ni B., Xie L. (2012). k-carrageenan sodium alginate beads and superabsorbent coated nitrogen fertilizer with slow-release, water-retention, and anticompaction properties. Ind. Eng. Chem. Res..

[36] Schneider C.A., Rasband W.S., Eliceiri K.W. (2012). NIH Image to ImageJ: 25 years of image analysis. Nat. Methods.

[37] Remunan-Lopez C., Bodmeier R. (1997). Mechanical, water uptake and permeability properties of crosslinking chitosan glutamate and alginate films. J. Control. Release.

