# Supplementary material for: Shellac Gum/Carrageenan Alginate-Based Core–Shell Systems Containing Peppermint Essential Oil Formulated by Mixture Design Approach
Source: Gels. 2021 Oct 3;7(4):162. doi: 10.3390/gels7040162 (PMC8544295; doi:10.3390/gels7040162)
Supplement: Supplementary file 1 [file gels-07-00162-s001.zip › gels-1382476-supplementary.pdf]

Supplementary Materials

Shellac Gum/Carrageenan Alginate-Based Core–Shell Systems Containing Peppermint Essential Oil Formulated by Mixture Design Approach

Andrea Foglio Bonda [1], Alessandro Candiani [1], Martina Pertile [1], Lorella Giovannelli [1] and Lorena Segale [1,*]

[1] Department of Pharmaceutical Sciences, Università del Piemonte Orientale, Largo Donegani 2/3, 28100 Novara, Italy
* Correspondence: lorena.segale@uniupo.it; tel. 0039 0321 375862.

4. Materials and Methods

2.4. Morphology and dimensions

Supplementary Tables

Table S1. TModels and corresponding equation used in the statistical analysis.

Model	Equation
Linear	$Variable = b_1ALG + b_2\mathrm{CGR} + b_3SHL$
Special Cubic	$Variable = b_1ALG + b_2\mathrm{CGR} + b_3SHL + b_{12}ALG \cdot CRG + b_{13}ALG \cdot SHL + b_{23}CRG \cdot SHL + b_{123}ALG \cdot CRG \cdot SHL$
Quadratic	$Variable = b_1ALG + b_2\mathrm{CGR} + b_3SHL + b_{12}ALG \cdot CRG + b_{13}ALG \cdot SHL + b_{23}CRG \cdot SHL$

Table S2. Coefficients calculated for each significative model.

Equation Coefficient	Variable				
	SF Wet	SF DRY	Diameter dry	Weight unit dry	Swelling
b1	0.7791	0.7958	1.76	2.61	33.99
b2	0.6863	0.6759	1.91	7.89	3,627.48
b3	0.8887	0.8278	1.83	5.25	95.03
b12	n.a.	n.a.	0.2760	-7.02	-4,367.59
b13	n.a.	n.a.	- 0.2461	-4.43	-204.78
b23	n.a.	n.a.	4.90	1.72	-3,873.42
b123	n.a.	n.a.	-8.03	n.a.	n.a.

Supplementary Figures

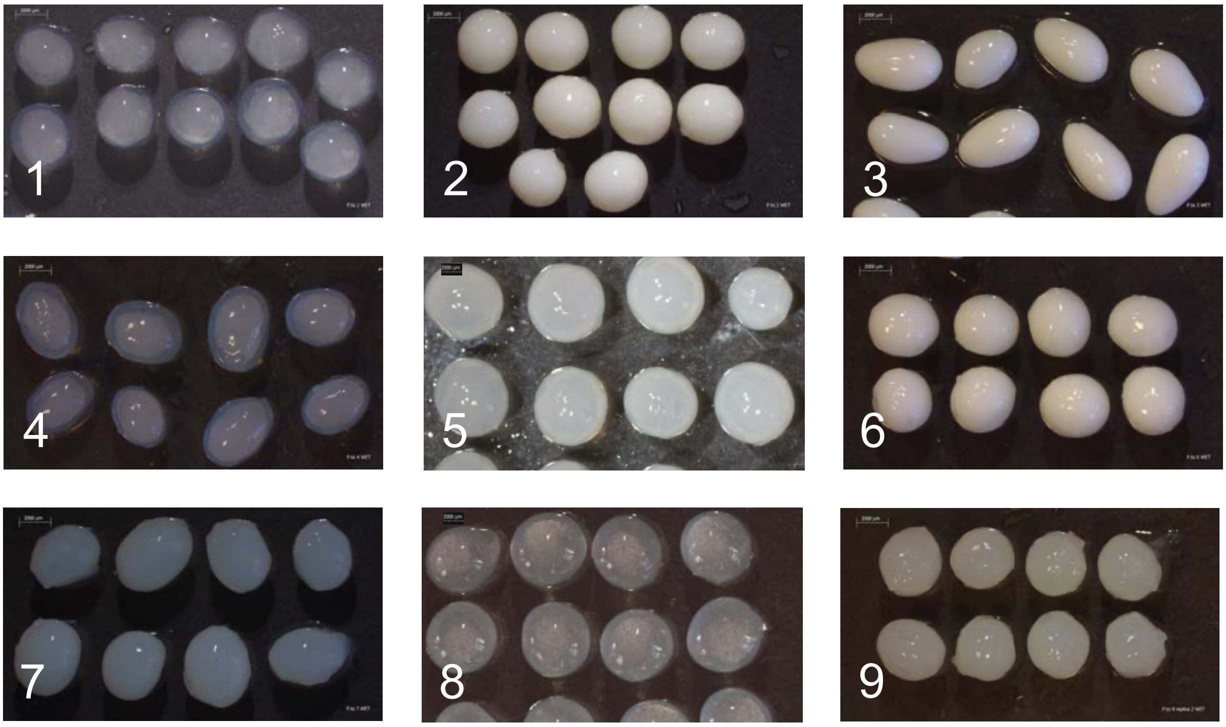

Figure S1. Detailed images of the wet core-shell systems (numbers correspond to the Experimental Points).

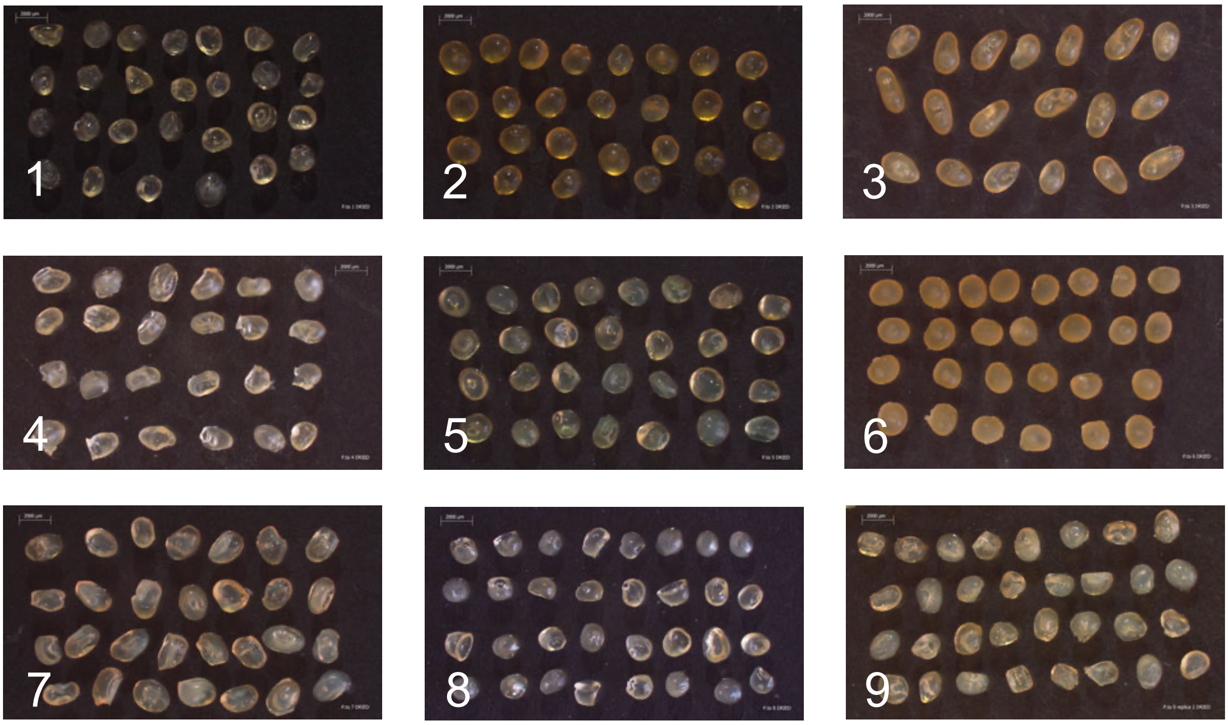

Figure S2. Detailed images of the dried core-shell systems (numbers correspond to the Experimental Points).
